# A metabolic perspective on microglia in Alzheimer’s disease

**DOI:** 10.4103/NRR.NRR-D-25-01035

**Published:** 2026-01-02

**Authors:** Moris Sangineto, Gaetano Serviddio

**Affiliations:** Internal Medicine, Department of Medical and Surgical Sciences, University of Foggia, Foggia, Italy

Alzheimer’s disease (AD) is the most common form of dementia, with 50 million people affected worldwide in 2019. With the continuous ageing of populations, the number of AD cases is expected to rise to approximately 150 million by 2050 (Sangineto et al., 2023). Given its significant impact on healthcare systems and society, novel biomarkers and therapeutic approaches are urgently needed.

Histopathology of AD brains is characterized by atrophy, plaques of amyloid-beta (Aβ) peptide aggregates, neurofibrillary tangles (mostly tau protein), loss of neurons and synapses, and dystrophic neurites. Recently, the role of microglia in AD pathogenesis has progressively emerged as crucial, although, in 1995, Alzheimer et al. already stated, “The glia have developed numerous fibers.” While microgliosis and astrogliosis are widely observed across various neurodegenerative disorders, each with distinct underlying causes, the precise role of these glial responses remains controversial: do they serve a protective function, contribute to disease progression, or are they merely incidental byproducts of neurodegeneration?

Microglia play a pivotal role in innate and adaptive immune response of the brain to pathogens as well as in maintaining homeostasis by removing apoptotic cell remnants and other extracellular detrimental products, hence controlling the neuronal microenvironment.

In a healthy brain, microglia live in a quiescent state, while they pass to activation in response to tumours, lesions, barrier dysfunction, and neurodegenerative diseases. Recent evidence indicates neuroinflammation and excessive microglial activation as the driving force of AD development. In the last decades, studies on immunometabolism highlighted that immune functions pass through specific programs of metabolic rewiring. For instance, during acute stimuli (e.g., lipopolysaccharide [LPS] and Aβ), microglia undergo a metabolic reprogramming characterized by a switch from oxidative phosphorylation to glycolysis via mammalian target of rapamycin (mTOR)–hypoxia-inducible factor 1-alpha (HIF-1α) signaling (Sangineto et al., 2023, 2024). This entails microglial pro-inflammatory activation with cytokine production and disease progression.

It is widely known that certain mutations are clearly associated with sporadic familial AD. Moreover, genome-wide association studies have identified several other risk factors also for late onset AD. Interestingly, most of these genes codify for receptors, which are preferentially or predominantly expressed on microglial cells and are directly or indirectly involved in cellular metabolism changes.

Yu and Ye (2015), in a review paper, finely described the landscape of receptors involved in Aβ recognition from microglia, underlining that some receptors, including CD36, RAGE, formyl peptide receptor 2 (FPR2), and Toll-like receptors 2/4 (TLRs2/4), are also involved in a consequent cytokine response. While our aim is not to provide another review on microglial Aβ phagocytosis, we emphasize that Aβ removal and neuroinflammation are interconnected processes depending on different bioenergetic states, and both these aspects should be considered simultaneously in AD research.

Several recent studies have demonstrated that Aβ production and its impaired clearance promote a hyper-inflammatory activation of microglia with consequent production of cytokines such as interleukin-1β and tumor necrosis factor-α, and reactive oxygen species generation, exacerbating neuronal damage. Notably, Aβ seems to act as a TLR4 activator, hence triggering proinflammatory responses (Paudel et al., 2020).

We have recently demonstrated that exposing human microglia to Aβ induces a pro-inflammatory and bioenergetic profile very similar to that observed with LPS. This observation was further confirmed as the co-stimulation with TAK242, a specific TLR4 inhibitor, abrogates the Aβ-activating effects (Sangineto et al., 2023). In accordance to this, TLR4 activation has been widely associated to AD brains both in humans and mice and, in murine models, the treatment with TAK242 provided neuroprotection and improvement of learning and memory functions (Cui et al., 2020). However, it is important to note that Aβ-induced inflammatory/metabolic activation of microglia only partially replicates the typical LPS-induced profile. Accordingly, bioenergetic repurposing and inflammatory activity observed in microglia of 3×Tg-AD mouse brains are considerably high, suggesting that additional factors contribute to TLR4-mediated inflammation in AD. LPS or other pathogen-associated molecular patterns/damage-associated molecular patterns might play a crucial role in microglia hyperactivity, as high levels of LPS have been found in both the brain and blood of AD patients (Sangineto et al., 2023).

Another key AD risk factor is apolipoprotein E (APOE), especially with its variant APOEε4, which is the principal risk factor for late onset AD. Beyond its typical function of lipid transport, APOE interacts with TLR4, facilitates Aβ uptake, and its expression is particularly elevated in AD brains, highlighting that a complex receptor activity is decisive in microglial function of Aβ clearance (Lee et al., 2023). We have recently analyzed the differential expression of genes in microglia isolated from 5×FAD mice *versus* WT mice at the age of 6 months, when inflammation is well represented in AD brains. Intriguingly, the most up-regulated gene was *APOE* (Sangineto et al., 2023).

In accordance with this, Lee et al. (2023) recently published data derived from single-cell, bulk, and spatial transcriptomics, demonstrating a potential link between APOE genotype and the regulation of microglial metabolism. APOEε4 microglia displayed a higher response to Aβ and LPS with metabolic reprogramming HIF-1α-mediated, characterized by tricarboxylic acid cycle disruption and hyper-glycolysis (Lee et al., 2023). This perfectly aligns with our results, since metabolic and pro-inflammatory activation in human microglia, with LPS or Aβ, was HIF-1α-mediated. Moreover, the inhibition of succinate dehydrogenase, a potent HIF-1a activator in macrophages, and the inhibition of HIF-1a itself with PX-478 abrogated glycolytic enhancement and cytokine production (Sangineto et al., 2023).

Another key regulator of metabolic impairments in microglia is the triggering receptor expressed on myeloid cells 2 (TREM2), whose genetic variants have been recently linked to AD. TREM2 is highly expressed in the brain by microglia as well as in certain peripheral tissues during inflammatory conditions. With TYRO protein tyrosine kinase binding protein (DAP12) interaction, TREM2 initiates the transduction of signals leading to chemotaxis, phagocytosis, and cell proliferation (Cai et al., 2022). It is indeed not singular that TREM2 loss-of function mutations entail a higher risk to develop AD, and transgenic models for TREM2 exhibit Ab plaque accumulation (Cai et al., 2022). These observations are also associated with microglia metabolic rearrangement. In TREM2-deficient microglia, cholesterol removal from myelin debris is impaired with consequent accumulation of cholesterol esters, while TREM2-deficient mice show mitochondrial dysfunction and defective phagocytosis in microglia (Cai et al., 2022). Moreover, AD risk genetic variants of TREM2 have shown reduced respiratory capacity and glycolytic switch failure with consequent impairment of Aβ phagocytosis (Piers et al., 2020).

This is interesting as we have described how certain genetic variants predispose microglia to hyper-metabolic phenotype and consequent hyper-inflammation, while metabolic defects induce microglia non-response and Aβ accumulation. Both conditions are associated with disease progression.

Along these lines, it becomes relevant to underline that APOE interacts with TREM2 (Cai et al., 2022; Lee et al., 2023), but also with TLR4; hence genetic variants impact on receptor activity, facilitating inflammation or impairing Aβ clearance.

The latter observation pushed some authors to propose microglia reactivation as a potential solution to remove amyloid plaque, again via metabolic manipulation (Baik et al., 2019; Lepiarz-Raba et al., 2023). Baik et al. (2019) revealed metabolic defects in the microglia of 5×FAD mice, stating that microglia, once activated, reach a chronic phase of tolerance with low cytokine response and inefficient phagocytosis. Stimulating microglia with interferon-γ, they were able to reactivate cells, via HIF-1α, increasing glycolysis and restoring phagocytosis with amyloid depletion (Baik et al., 2019). At this point, we should ask what the most appropriate strategy is, as current studies appear to follow two opposing approaches: targeting microglia-derived inflammation or enhancing microglial phagocytic function.

**Figure 1:**
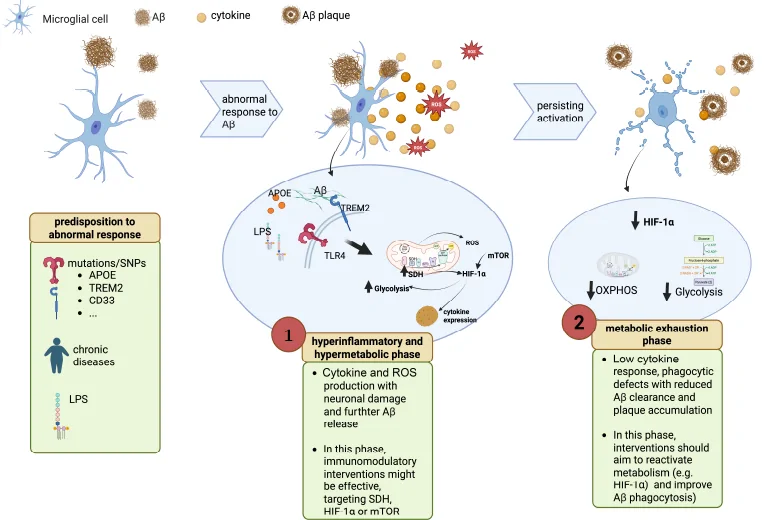
Two-phase metabolic hypothesis of microglial role in Alzheimer’s disease. Microglia carrying genetic or environmental risk factors (e.g., APOE, TREM2, CD33 variants, and chronic inflammatory stimuli) display an abnormal response to amyloid-β (Aβ). In the early hyperinflammatory/hypermetabolic phase (1), Aβ and lipopolysaccharide (LPS) activate TLR4/TREM2 pathways, driving mTOR–HIF-1α–dependent glycolysis, succinate dehydrogenase (SDH) activation, reactive oxygen species (ROS) generation, and cytokine release, which promote neuronal damage and further Aβ deposition. With persistent stimulation, microglia enter a metabolic exhaustion phase (2) characterized by reduced HIF-1α activity, impaired oxidative phosphorylation (OXPHOS) and glycolysis, low cytokine output, defective phagocytosis, and plaque accumulation. These sequential metabolic states identify two therapeutic windows: early interventions targeting SDH, HIF-1α, or mTOR to limit inflammation, and later strategies aimed at reactivating microglial metabolism and Aβ clearance. Aβ: Amyloid-β; APOE: apolipoprotein E; HIF-1α: hypoxia-inducible factor 1-alpha; LPS: lipopolysaccharide; mTOR: mammalian target of rapamycin; OXPHOS: oxidative phosphorylation; ROS: reactive oxygen species; SDH: succinate dehydrogenase; TLR4: Toll-like receptor 4; TREM2: triggering receptor expressed on myeloid cells 2.

We believe that the root of the problem should be observed from another perspective. In our recent work, we have detected a hypermetabolic state in early stages, when the brain of 3×Tg-AD mice shows high levels of inflammation (Sangineto et al., 2023). At this time point, microglia metabolism can be manipulated, reducing inflammation. In the later stages, cytokine production lowers and microglia display metabolic defects, shaping the “tolerant” phase described by Baik et al. (2019), characterized by a sort of “metabolic exhaustion” (**Figure 1**).

Therefore, it is likely that both observations are valid, profiling a “two-phase metabolic hypothesis” for AD pathophysiology, and indicating two distinct therapeutic targets: hyperinflammation in the early stages and phagocytic defects in the later stages (**Figure 1**).

From this perspective, the *primum movens* in AD is characterized by a dysregulated microglial response to Aβ rather than an aberrant production of it.

Lee et al. (2023) have recently centered the point, demonstrating that APOE4 promotes a receptor activity that leads to excessive microglia metabolic activation at early phases with consequent inflammatory neuronal damage, and metabolic exhaustion at latter phases with defective Aβ clearance. Beyond the genetic predisposition, it is deducible that metabolic diseases such as diabetes and obesity might represent important AD risk factors because of their intrinsic interference with bioenergetic and immunometabolic homeostasis.

In conclusion, AD should be considered a continuum of events, progressing from Aβ production to microglial activation, hyperinflammation, microglial exhaustion, and ultimately impaired Aβ clearance (**Figure 1**).

Metabolic reprogramming is the way by which microglia regulate its activity and are probably the keyhole for researchers to observe microglia abnormal functions, but also to offer concrete solutions in the next future.

The repeated failure of clinical trials in AD likely reflects the oversimplified focus on single-target interventions. A paradigm shift toward stage-specific therapeutic strategies is needed, with future studies aligning treatment approaches to the dynamic evolution of AD pathology.
